# Deaths Registration and Burials Bill

**Published:** 1908-09-26

**Authors:** Jas. R. Williamson

**Affiliations:** 100 Chedington Road, Upper Edmonton, London, N.


### Editor's Letter-Box.

**Our correspondents are reminded that prolixity is a great bar to publication, and that brevity of style and conciseness of statement greatly facilitate early insertion.**

### DEATHS REGISTRATION AND BURIALS BILL.

(*To the Editor of* The Hospital.)

Sir,—A Bill of this title, which, though it does not go all the way advocated by the Association for the Prevention of Premature Burial during the past thirteen years, will yet make the occurrence of these underground tragedies less easy than they now are, was ordered by the House of Commons to be printed on July 20, and receives the approval of this Association as an instalment of the necessary legislative precautions. The Bill consists of sixteen clauses, is presented by Mr. George Greenwood, supported by Sir Walter Foster, Mr. John Robertson, Mr. Smeaton, Dr. Rutherford, Mr. Hart-Davies, Dr. Shipman, and Mr. Atherley-Jones. Amongst its provisions may be mentioned (1) Prohibition of registry of death where no proper certification of death has taken place. (2) Qualified informant to state to registrar whether a medical practitioner was in attendance on deceased person. (3) Duty of medical practitioner who attended deceased person to send death certificate to the registrar. (4) Provisions for cases where no medical practitioner has been in attendance on a deceased person. (5) Provisions as to verification of death where no inquest is held. (6) Provisions in case of death not due to disease. (7) Appointment and duties of public certifier of deaths. (8) Prohibition of burial except after registration of death and in accordance with certificate or order authorising burial. (9) Amendment of law as to orders and certificates authorising burials. (10) Registration of still births. (11) Forms of certificates. Under this clause provision is made that the death certificate shall be so framed as to state (*a*) that the person by whom the certificate is signed has inspected the body; (*b*) that death has taken place, and the signs from which the fact of death is inferred; (*c*) the cause of death; and (*d*) that the person by whom the certificate is signed was the registered medical practitioner in attendance on the deceased during his last illness, or the public certifier of deaths, as the case may be. (12) Powers of Local Government Board and of Registrar-General to make regulations. (13) Sending and signing and filling up of certificates, etc. (14) Penalties for not sending certificates, etc., and for false certification, etc. (15) Interpretation of expressions used in the Bill, such as "registrar," "burial authority," etc.

It is hoped that this useful non-party Bill may receive the support of members of Parliament of all shades of opinion. If any of your readers are in doubt as to the necessity of legislation for the prevention of premature burial, as well as of crime, I should be pleased to send pamphlets on the subject on receiving a stamped addressed envelope.

I am, sir, your obedient servant,

Jas. R. Williamson.

100 Chedington Road, Upper Edmonton, London, N.,
September 2, 1908.

